# Intrafraction Prostate Motion Management During Dose-Escalated Linac-Based Stereotactic Body Radiation Therapy

**DOI:** 10.3389/fonc.2022.883725

**Published:** 2022-04-07

**Authors:** Denis Panizza, Valeria Faccenda, Raffaella Lucchini, Martina Camilla Daniotti, Sara Trivellato, Paolo Caricato, Valerio Pisoni, Elena De Ponti, Stefano Arcangeli

**Affiliations:** ^1^ Medical Physics Department, ASST Monza, Monza, Italy; ^2^ School of Medicine and Surgery, University of Milan Bicocca, Milan, Italy; ^3^ Department of Physics, University of Milan, Milan, Italy; ^4^ Radiation Oncology Department, ASST Monza, Monza, Italy

**Keywords:** prostate cancer, Steretactic Body Radiation Therapy (SBRT), extreme hypofractionation, Image-guided Radiation Therapy (IGRT), intrafraction motion mitigation, real-time electromagnetic tracking

## Abstract

**Background:**

Extreme hypofractionation requires tight planning margins, high dose gradients, and strict adherence to planning criteria in terms of patient positioning and organ motion mitigation. This study reports the first clinical experience worldwide using a novel electromagnetic (EM) tracking device for intrafraction prostate motion management during dose-escalated linac-based stereotactic body radiation therapy (SBRT).

**Methods:**

Thirteen patients with organ-confined prostate cancer underwent dose-escalated SBRT using flattening filter-free (FFF) volumetric modulated arc therapy (VMAT). The EM tracking device consisted of an integrated Foley catheter with a transmitter. Patients were simulated and treated with a filled bladder and an empty rectum. Setup accuracy was achieved by ConeBeam-CT (CBCT) matching, and motion was tracked during all the procedure. Treatment was interrupted when the signals exceeded a 2 mm threshold in any of the three spatial directions and, unless the offset was transient, target position was re-defined by repeating CBCT. Moreover, the displacements that would have occurred without any intrafraction organ motion management (i.e. no interruptions and repositionings) were simulated.

**Results:**

In 31 out of 56 monitored fractions (55%), no intervention was required to correct the target position. In 25 (45%) a correction was mandated, but only in 10 (18%), the beam delivery was interrupted. Total treatment time lasted on average 10.2 minutes, 6.7 minutes for setup, and 3.5 minutes for beam delivery. Without any intrafraction motion management, the overall mean treatment time and the mean delivery time would have been 6.9 minutes and 3.2 minutes, respectively. The prostate would have been found outside the tolerance in 8% of the total session time, in 4% of the time during the setup, and in 14% during the beam-on phase. Predominant motion pattern was posterior and its probability increased with time, with a mean motion ≤ 2 mm occurring within 10 minutes.

**Conclusions:**

EM real-time tracking was successfully implemented for intrafraction motion management during dose-escalated prostate SBRT. Results showed that most of the observed displacements were < 2 mm in any direction; however, there were a non-insignificant number of fractions with motion exceeding the predefined threshold, which would have otherwise gone undetected without intrafraction motion management.

## Background

Clinical results from retrospective studies allowed to formulate the hypothesis that the linear quadratic α/β ratio of prostate cancer is generally lower than in the majority of other human tumors (estimated to ~ 1.5 Gy) (1–3). Based on this strong radiobiologic rationale, various trials (4–7) showed that prostate cancer could benefit from hypofractionated regimens of Radiation Therapy (RT). Along with huge advances in radiation technology that have permitted improved precision in radiation delivery and increased protection of the organs at risk (OARs), extreme hypofractionation using Stereotactic Body Radiation Therapy (SBRT) has also been explored with optimal results in terms of biochemical control and side effects (8), becoming the standard of care treatment option of low-intermediate risk prostate cancer (9). The findings from two large systematic reviews (10, 11) and of the one phase III study, HYPO-RT-PC (12), established the most compelling evidence in favor of extreme hypofractionation, while the efficacy data for the PACE-B trial (13) are still pending.

Due to the inherent dose per fraction escalation and the low number of fractions used, SBRT necessitates high dose gradients to be employed with tighter margins than conventional treatment. Therefore, errors in actual dose delivery precision and accuracy can lead to inadequate target coverage and/or overdose of surrounding OARs. The major drawback remains the significant and unpredictable intrafraction prostate motion, mainly due to rectal and bladder filling (14–18). Without continuous monitoring and intervention, in approximately 10% of patients, intrafractional motion would lead to target missing (19). The Calypso System (Varian Medical Systems, Palo Alto, CA), which uses 3 radiofrequency beacons implanted in the prostate to localize and monitor its motion in real-time (20–22), is an example of different methods for imaging, tracking, and correcting for prostate displacements during treatment delivery. Despite its proven accuracy, the Calypso system is an invasive technique for the patient, and the severe artifacts on Magnetic Resonance Imaging (MRI) caused by the beacons could impact treatment planning and radiological follow-up assessments.

A novel electromagnetic (EM) transmitter-based device without surgical intervention to localize and monitor the prostate and the urethra was implemented in the first clinical use worldwide. This study reports the results of tracking in real-time the organ motion during dose-escalated linac-based SBRT for organ-confined unfavorable prostate tumors.

## Methods

### Patient Setup and Treatment Planning

Patient population and treatment planning protocol have been described previously (23). Briefly, patients were immobilized in the supine position with arms over their chest using the FeetFix system (CIVCO Medical Solutions, Iowa, US) attached to the couch for ankle fixation. The bladder was filled with 100 cc of saline solution *via* a 16 French Foley catheter during the simulation and a rectal micro-enema was administered. Same bladder and bowel preparation was repeated for each fraction to assess anatomical reproducibility and limit prostate mobility. No rectal immobilization or rectal spacer devices were adopted. To properly delineate the target volume and the OARs, a non-contrast enhancement computed tomography (CT) and a T2-weighted 3D MRI scans were acquired and fused. The planning target volume (PTV) consisted of the prostate gland and the seminal vesicles with a 2 mm isotropic expansion. A margin of 2 mm was applied around the catheter to calculate a planning organ at risk volume (PRV) for the urethra and to enable significant dose-sparing at this level, by allowing a negative dose-painting in order to reduce the risk of treatment-related urinary toxicity.

The treatment schedule consisted of 40 Gy in 5 fractions or 38 Gy in 4 fractions delivered consecutively over one week. With an α/β ratio of 1.5 Gy, the corresponding Biologically Effective Dose (BED) was 253 Gy and 279 Gy, respectively. Treatment was planned with volumetric modulated arc therapy (VMAT) using typically two 10 MV flattening filter-free (FFF) arcs on a VersaHD linear accelerator (Elekta AB, Stockholm, Sweden). Plans were optimized using penalties and priorities to have the 95% isodose covering at least 95% of the PTV and to fulfill the dose-volume constraints to OARs, such as bladder, PRV of urethra rectum, rectum wall, and penile bulb, and were calculated with the Monte Carlo algorithm (1 mm grid spacing and 1% statistical uncertainty for calculation) of Monaco Treatment Planning System (Elekta AB, Stockholm, Sweden).

### Intrafraction Motion Tracking and Intervention

The intrafraction organ motion evaluation was performed by RayPilot System (24, 25) (Micropos Medical AB, Gothenburg, Sweden), a novel real-time EM tracking device. The system consists of a wired transmitter, that is integrated into a dedicated lumen of the RayPilot HypoCath, a Foley catheter inserted into the patient, and the RayPilot Receiver, a platform that is placed on the existing carbon fiber couch under the patient. The transmitter, consisting of a choke coil (diameter 3 mm, length 11 mm) and a cable, is connected to the receiver plate during each fraction to activate the device. An antenna array captures the signal sent by the transmitter, and the position of the transmitter is located. The system was calibrated to the treatment room isocenter and allowed for treatment localization as well as motion tracking. The position is given along the three-dimensional axes (lateral, longitudinal, and vertical) at a sampling frequency of 30 Hz. Rotations around the vertical axis (yaw) and the lateral axis (pitch) are also detected by the system. Treatment couch bending due to patient weight was measured and considered in the system.

**Figure 1:**
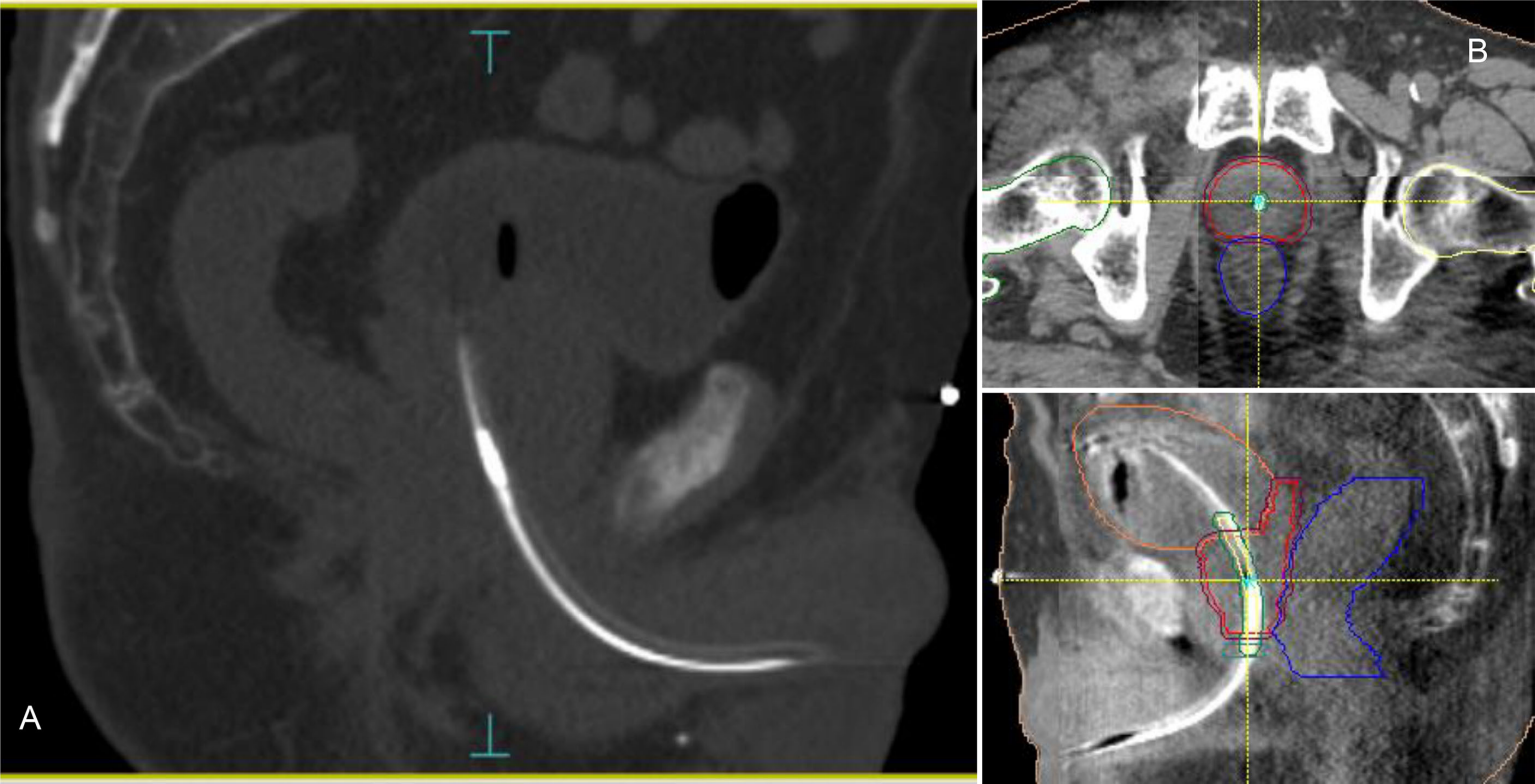
RayPilot HypoCath. The transmitter choke is visible inside the urinary catheter within the prostatic urethra **(A)**. Planning CT to daily CBCT matching: proper rectum and bladder filling verification, in addition to transmitter and urethra localizations; in case of deformation or deviation of the urethral path, the catheter was placed inside the urethra PRV along with the entire extension of the prostate **(B)**.

Accurate patient setup was achieved by a ConeBeam-CT (CBCT) soft tissue matching prior to treatment (**Figure 1**). Motion tracking was enabled immediately after the start of the CBCT acquisition by setting the initial position detected by the system equal to zero. A shift in the transmitter position was used as a surrogate for the prostate motion. Due to the demand for a very accurate delivery in such treatments, the beam delivery was promptly interrupted every time a shift of the transmitter exceeded more than 2 mm from its planned position in any of the three spatial directions. In case of prolonged drift outside this tolerance (15 seconds), a new CBCT was acquired and matched and the couch position corrected for taking into account the prostate motion before resuming the beam. Anytime a CBCT acquisition was mandated, a new RayPilot position was set in the system to get a new starting point with respect to which displacements were calculated and shown. Because the prostate may also move between the initial target positioning procedure and the beam-on time, this real-time tracking system allowed to detect and correct any possible target displacement observed in the setup phase according to the aforementioned rules. With conventional Image Guided Radiation Therapy (IGRT), this shift would have gone unnoticed and not accounted for.

### Data Processing and Analysis

Real-time measurement of the transmitter displacement was recorded for each treatment fraction. After the treatment, the log files including the transmitter positions and beam-on indications were exported with an update rate of 15 Hz in XML format. Intrafraction motion was calculated by computing prostate shifts for the translational and rotational axes relative to the initial zero position. A C++ program was developed for the analysis of the data files produced by the tracking system software; ROOT data analysis framework libraries were exploited for the graphical representation of target translational and rotational deviations. The main objective of the program elaboration was to automate as much as possible the analysis procedure, minimizing the required user actions. Treatment sessions were analyzed with and without beam gating and motion correction interventions. Real prostate motion data (i.e. with no interruptions and repositioning included) were obtained by removing all changes due to the reset of the transmitter position with the acquisition of a new CBCT. Moreover, the trajectories that would have occurred without any organ motion management and beam gating were simulated by adjusting setup and delivery duration. A fixed duration of 3.5 minutes was used to include the time for the CBCT acquisition and the registration to the reference planning CT. For the delivery, the real delivery time of the specific treatment plan without interruptions was used.

## Results

The localization uncertainty of the RayPilot System, measured in a precision test procedure, was 0.34 ± 0.18 mm [radial mean ± standard deviation (SD)]. The procedure consisted of calculating the radial error in 4 displacements from the calibration center point (i.e. 2 longitudinal and 2 vertical and lateral diagonal positions on each side of the center point, respectively), assessed in both laboratory and clinical environment.

Overall, 56 treatment fractions were delivered and analyzed, and 84 CBCT to planning CT matchings were performed. In 31 sessions, corresponding to 55% of the total, the signal remained within the predefined tolerance for the whole treatment time and no intervention was required to correct the target position as a result of an excessive prostate movement. Only in 3 of those cases (5%), the target moved out of the 2 mm threshold, but it promptly returned within the safety threshold. In the other 25 sessions (45%) the prostate exceeded the tolerance after the initial CBCT verification. In 10 cases (18%) a non-re-entering prostate shift occurred during the treatment delivery, requiring a beam interruption and a new CBCT.

Considering all fractions, the median duration from the start of the EM tracking procedure to the end of the delivery was 8 minutes, with an average time of 10.2 ± 4.2 minutes (range 5.5 - 22.7), 6.7 ± 3.8 minutes (range 2.7 - 17.8) for patient setup and 3.5 ± 0.9 minutes (range 2.5 - 7.3) for beam delivery (beam-on time + interruptions). Noteworthy since the intervention procedure in halting the beam was manual, there was a lag between the alert and beam-off estimated in approximately 1 second, a small amount of the 150- to 250-second beam-on time. Without any intrafraction motion management, the overall mean treatment time and the mean delivery time would have been 6.9 minutes (range 5.5 - 9.9) and 3.2 minutes (range 2.5 - 4.2), respectively. The evaluation of the transmitter trajectories of the gated treatments has been described previously (22). Without any intrafraction motion management, (i.e. without beam gating and patient position corrections) the prostate would have been found outside the 2 mm tolerance in 8% of the total session time, namely in 4% during the setup time and in 14% during the beam delivery, respectively. **Table 1** illustrates the percentage of time that the prostate spent outside the 2 mm threshold in each of the three spatial directions during the setup, delivery, and total treatment either without or with the intrafractional organ motion management. The difference in time percentages between the two scenarios is also reported.

**Table 1:** Percentage of the setup time, delivery time, and total treatment time spent by the prostate outside the 2 mm threshold by spatial direction (LAT, lateral; LNG, longitudinal; VRT, vertical) without the intrafractional organ motion management and with the real-time management.

Time spent outside the 2 mm threshold	Setup			Delivery			Treatment		
	LAT	LNG	VRT	LAT	LNG	VRT	LAT	LNG	VRT
Without intrafraction motion management	0%	2%	3%	5%	9%	14%	2%	5%	8%
With intrafraction motion management	3%	5%	8%	1%	2%	4%	2%	4%	7%
Difference	-3%	-3%	-5%	4%	7%	10%	0%	1%	1%

**Figure 2:**
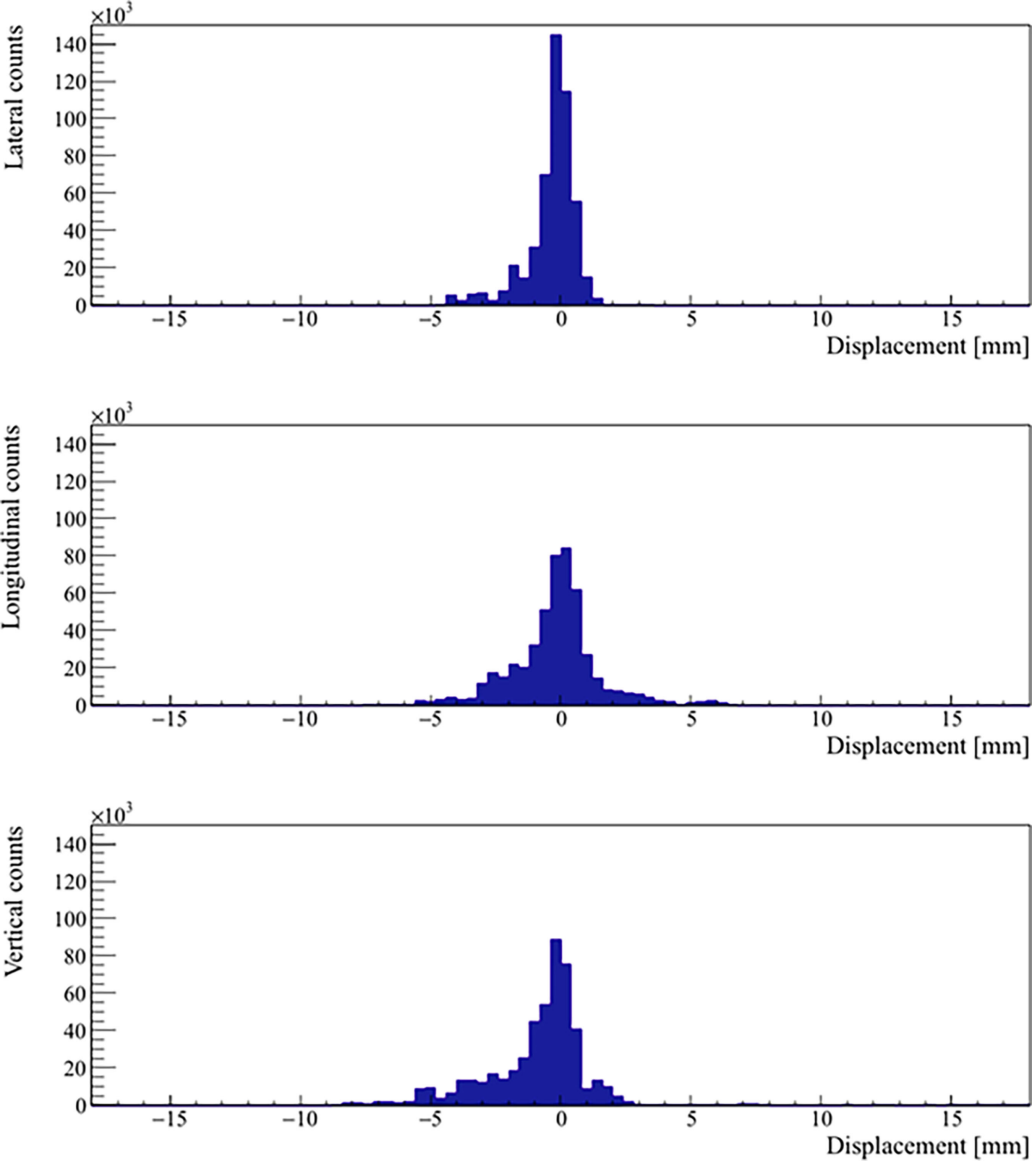
Distribution of the real prostate translational motion with no interruptions and patient position corrections. The positive axis represents a displacement in left, superior, and anterior directions, respectively.

**Figure 3:**
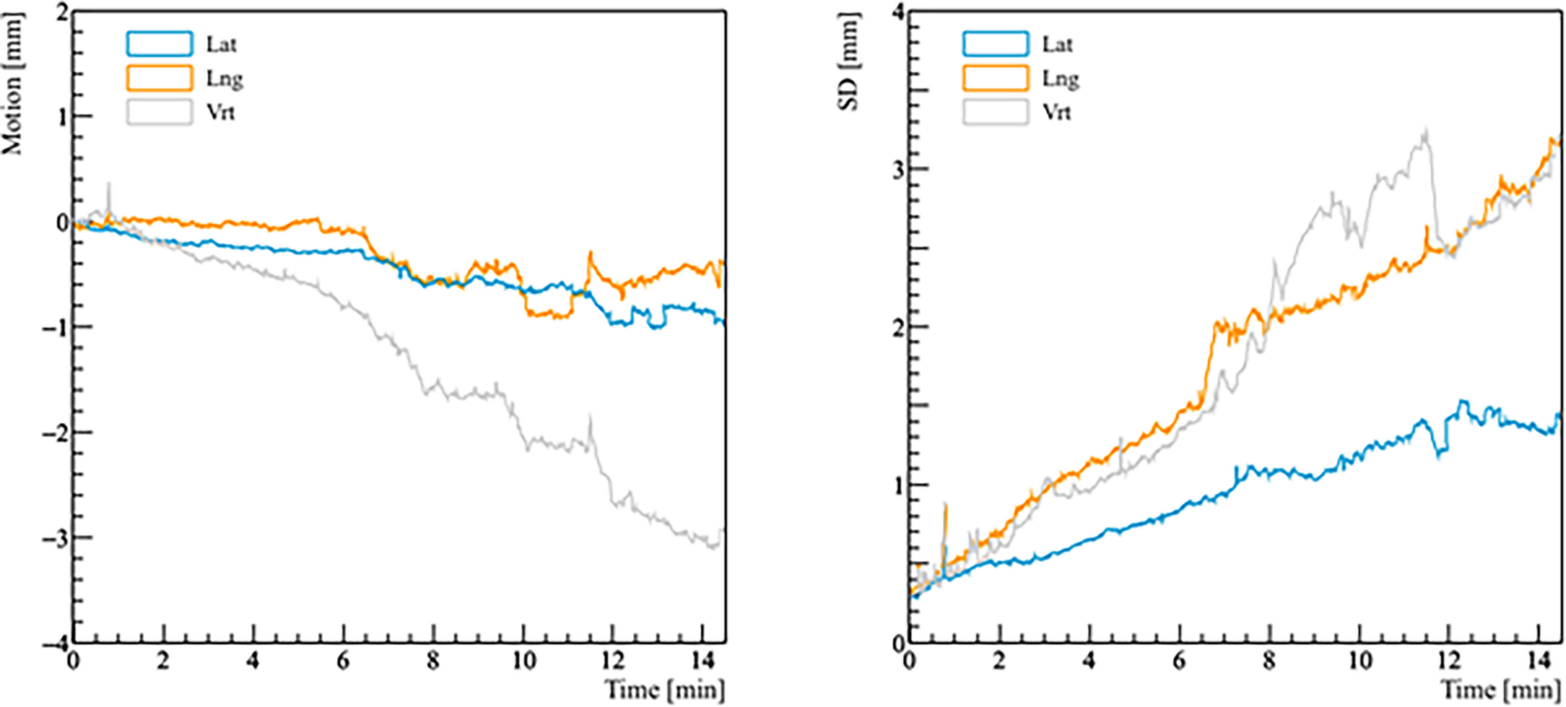
Real prostate motion with no interruptions and patient position corrections as a function of time (t = 0 at the beginning of the initial CBCT). The left panel shows the mean variations from the initial position of the prostate, the right panel shows the standard deviation (SD) of the mean motion.

Real prostate motion data analyzed from all the patients are presented in **Table 2**. Mean displacements in lateral, longitudinal, and vertical directions were < 1 mm, indicating that the overall motion occurred randomly. The vertical axis showed the higher mean value in the posterior direction and also the mean standard deviation was wider than in the other two directions. Mean absolute values of real prostate motion were found within our PTV margins, but the mean absolute maximum was not in two of the three axes. The prostate predominant displacements occurred in the inferior and posterior directions. It is also apparent from **Figure 2** that the distribution of the real prostate translational shift along the three directions was asymmetrical. A systematic drift in the mean prostate position to the right, inferiorly, and posteriorly was noticed. To confirm these data, real prostate motion was plotted as a function of treatment time, considering t = 0 at the beginning of the initial CBCT (**Figure 3**).

**Table 2:** Mean, standard deviation (SD), mean absolute, and mean max absolute of the real prostate translational data from all the 56 fractions with no interruptions and patient position corrections.

Direction	Mean (mm)	SD (mm)	Mean absolute (mm)	Mean max absolute (mm)
Lateral	-0.36	0.95	0.65	1.78
Longitudinal	-0.21	1.69	1.17	3.17
Vertical	-0.92	1.95	1.42	3.83

The negative sign represents a displacement in right, inferior, and posterior directions, respectively. Max deviation represents the absolute maximum displacement for time point in the 3 spatial directions.

**Figure 4:**
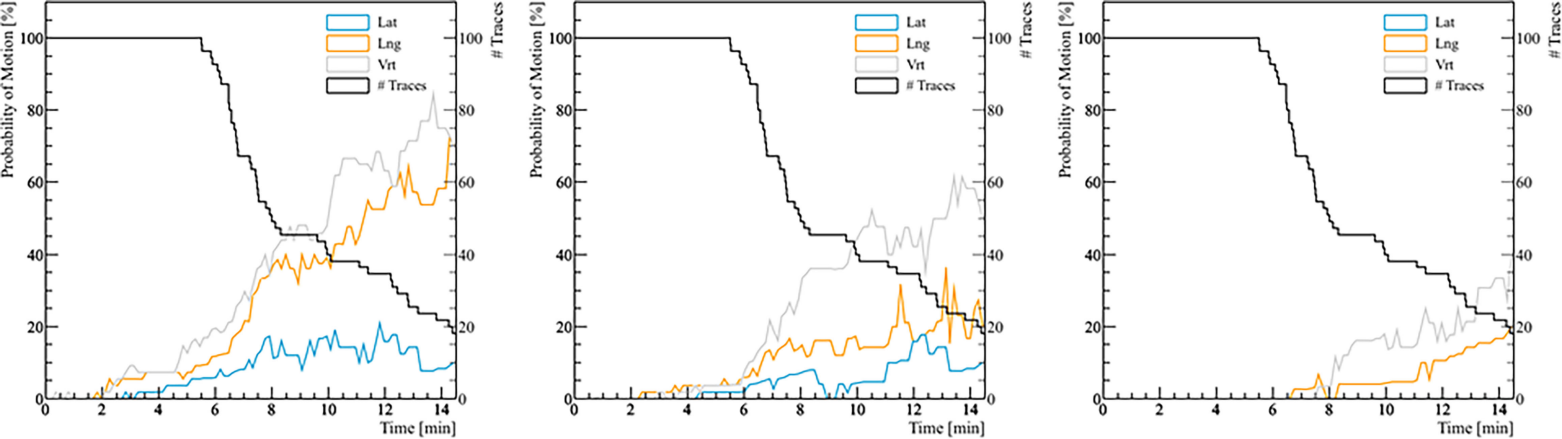
The probability of real prostate motion with no interruptions and patient position corrections as a function of time. The left panel shows the probability of motion > 2 mm, the middle panel for motion > 3 mm, and the right panel for motion > 5 mm. The black line represents the number of traces analyzed with respect to treatment time.

The analysis of the probability of real prostate motion as a function of time is shown in **Figure 4**. The probability of motion > 2 mm in the lateral, longitudinal, and vertical direction after 5 minutes was 3.6% (2/56), 8.9% (5/56), and 14.3% (8/56), respectively. Overall, half of the fractions were accomplished within 8 minutes. In that time, the same probability was 11.1% (3/27), 37.0% (10/27), and 40.7% (11/27) for the three directions. The probabilities of motion > 3 mm in lateral, longitudinal, and vertical direction after 5 and 8 minutes were 1.8% (1/56), 3.6% (2/56), 3.6% (2/56), and 7.4% (2/27), 14.8% (4/27), 33.3% (9/27), respectively. There were no fractions that had a prostate deviation > 5 mm in any direction after 5 minutes, while only 1 fraction out of 27 (3.7%) moved out of 5 mm posteriorly after 8 minutes.

**Figure 5:**
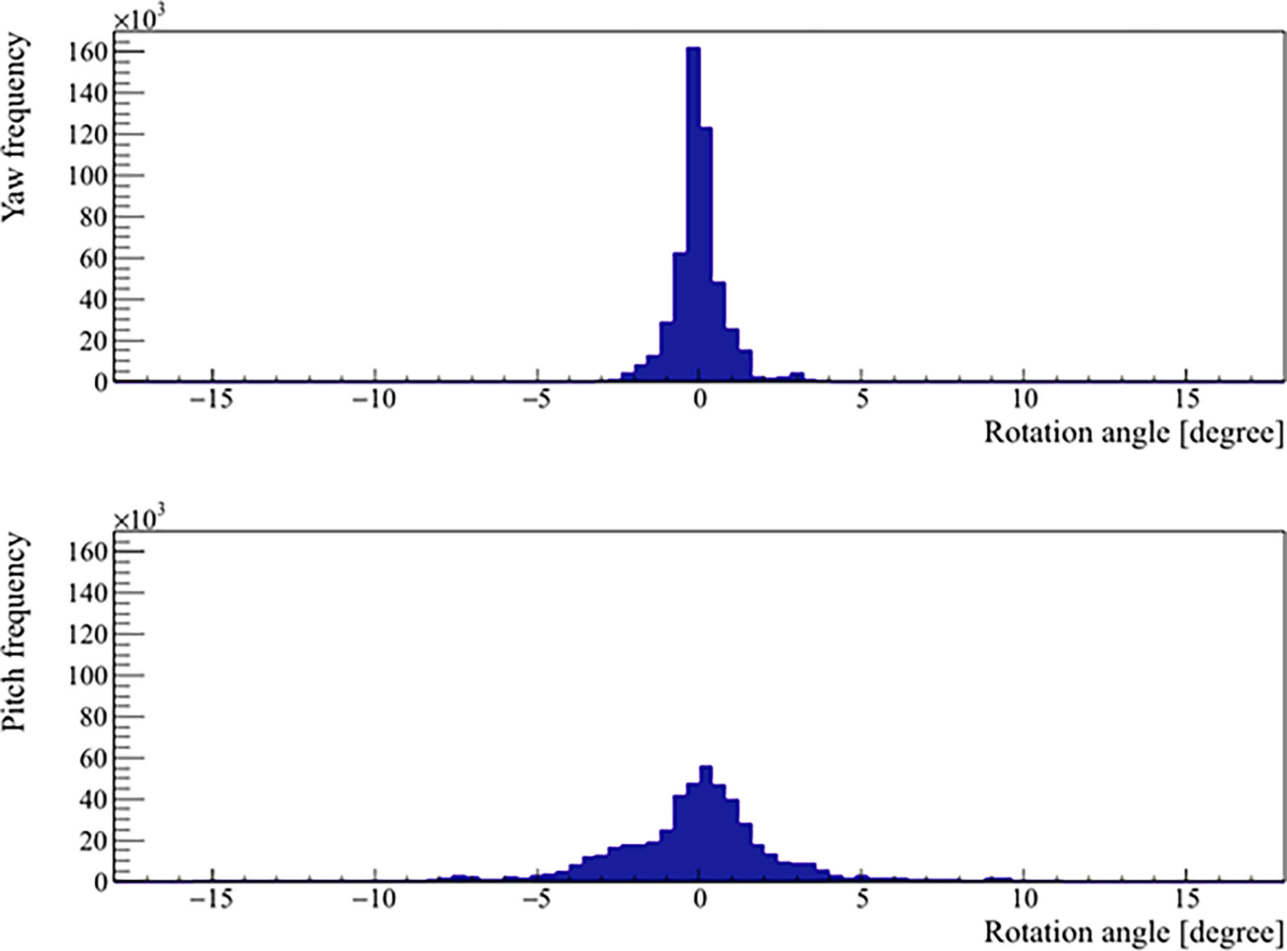
Distribution of the real prostate rotational variations with no interruptions and patient position corrections.

The mean, standard deviation, minimum, and maximum of the rotation angles determined from all the patients are shown in **Table 3**. In the pitch axis, a systematic rotation, which is absent in the yaw axis, was observed. Meanwhile, the range and standard deviation of rotation angles were larger in the pitch axis. The distribution of prostate rotation angles in the two axes, graphically represented in **Figure 5**, showed an asymmetric distribution to the negative axes and extreme rotations beyond 10 degrees in some instances.

**Table 3:** Mean, standard deviation, and range of the real prostate rotational variations with no interruptions and patient position corrections.

Axis	Mean angle	SD	Min angle	Max angle
Pitch	-0.2°	2.6°	-15.2°	11.0°
Yaw	0.0°	0.8°	-2.8°	3.6°

## Discussion

IGRT has been demonstrated to improve treatment accuracy and reduce side effects associated with prostate irradiation (26–28). In this study, intrafraction motion management was not the only strategy employed to assure the SBRT efficacy and an acceptable toxicity profile. Further refinements to aim at this purpose included strict bowel preparation, bladder filling, MR-based treatment planning with negative dose-painting around the urethra, and fast treatment delivery time with FFF VMAT beams. Although most patients experienced minimal motion during treatment, some fractions required beam interruptions to correct for prostate displacement. Indeed the 45% of treated fractions would have resulted in undetected displacements of more than 2 mm without intrafraction motion management. In the context of extreme hypofractionation, even a single fraction with unexpected organ motion can lead to potentially detrimental dosimetric and clinical consequences. The excellent early toxicity rates, compliance, and biochemical outcomes seen in the present series (23) suggest that treatment was delivered accurately and precisely.

Pretreatment orthogonal radiographs, CBCT, and/or ultrasound are commonly employed methods to accomplish interfraction motion management. These approaches are useful for verifying initial patient setup but are difficult to use in assessing intrafraction organ motion during treatment delivery. To continuously track the prostate during treatment, several commercially available techniques, including surface monitoring, kV and MV X-ray imaging-based methods, marker implantation, and real-time segmentation in kV and MV images, EM transponders or transmitters, ultrasound acquisitions, and MRI techniques, are now routinely used in practice (29). Several of them are expensive, requiring additional equipment unavailable on a standard LINAC. The most consistent example is the CyberKnife (CK) robotic radiosurgery system. The CK technique requires an invasive procedure by the positioning of fiducials within the prostate parenchyma. A complex X-ray imaging system that captures high-resolution images onto paired orthogonal amorphous silicon flat-panel detectors ensures target tracking (30). Our findings indicated that, even when the treatment was interrupted for prostate motion correction, the majority of fractions were delivered in less than 10 minutes. This is remarkable for an ultrahypofractionated treatment, especially in light of the significant amount of time of CK treatments, ranging from 20 to 90 minutes (31–33). Longer treatments may increase the risk of errors and patient discomfort, affecting the intrafraction motion and potentially reducing the clinical benefits associated with the use of a cutting-edge technology (34, 35).

Our measured intrafractional data on prostate real motion are similar to previously published observations (19, 36–40). The predominant motion was anteriorly-posteriorly, which is consistent with the current literature, although a not null value was detected also in the mean lateral displacement. From the analysis of the prostate motion as a function of treatment time, we showed that the probability of motion increased with time, with a mean real motion ≤ 2 mm within 10 minutes. Remarkably, Legge et al. (18) have noted translations as small as 0.01 ± 0.23 mm, 0.21 ± 0.12 mm, and 0.11 ± 0.64 mm in lateral, longitudinal, and vertical direction, respectively, with the incorporation of a rectal retractor device and real-time kV infraction monitoring. It should be noted that the calculated prostate real motion reflects a scenario in which intrafractional displacements are not corrected in real-time. With the integration of real-time intrafractional motion monitoring, the use of tighter than conventional margins (5 mm, 3 mm posteriorly) is conceivable with adequate target coverage. According to our findings, it could be argued that the vast majority of the patients would not have required intrafractional adjustment if the PTV margins were set up to 5 mm. However, with wider margins, it would not have been possible to escalate the dose while respecting the dose-volume constraints for the rectum and the bladder due to the increasing overlap between target volumes and organs at risk.

Additionally, we observed a minor asymmetry in the distribution of prostate rotations, particularly in the pitch axis. Pitch can be thought of as a tilt in the longitudinal plane and thus is the most strongly affected by any alterations in rectal volume. Our observed rotations were smaller than those observed by Wolf et al. (41). This may be due to our strict adherence to the empty bowel protocol prior to both planning and treatment, minimizing the rectal filling from the proximal direction. It has previously been reported that in plans optimized for motion robustness, clinical target volume D95 is insensitive to yaw and roll of up to 10°, but it’s more sensitive to pitch, which leads to poorer dosimetric results already at around 5° (42).

RayPilot System is a non-ionizing non-interfering real-time positioning system that has the advantage of being removed upon treatment completion, enabling MRI follow-up without any artifact, and does not require any permanent treatment room installations, thus providing a theoretical improvement over available options (20, 21). Furthermore, the introduction of the RayPilot HypoCath resulted in a less invasive and more stable device than the transperineal implanted wired transmitter (43, 44). However, since the absolute localization accuracy of the system may not be high enough for interfraction localization of the prostate, mostly due to the uncertain positional reproducibility of the catheter balloon with respect to the bladder wall, we recommend to combine real-time prostate motion monitoring by RayPilot with an independent IGRT system, and namely a volumetric one, to account for the optimal rectal and bladder filling.

## Conclusion

EM real-time tracking was successfully implemented for intrafraction motion management during dose-escalated prostate SBRT. Findings showed that most of the observed displacements were < 2 mm in any direction; however, there were a non-insignificant number of fractions with a motion exceeding the predefined threshold, which would have otherwise gone undetected without intrafraction motion management.

## Data Availability Statement

The raw data supporting the conclusions of this article will be made available by the authors, without undue reservation.

## Ethics Statement

Ethical review and approval was not required for the study on human participants in accordance with the local legislation and institutional requirements. Written informed consent for participation was not required for this study in accordance with the national legislation and the institutional requirements.

## Author Contributions

DP was the lead author, who participated in data collection, data analysis, manuscript drafting, table/figure creation, and manuscript revision while also aiding in study design. VF and RL contributed equally to this work and participated in data analysis, manuscript drafting, table/figure creation, and manuscript revision. MD organized and performed the analysis of the dataset. ST, PC, and VP participated in data collection and data analysis. EP is a senior author who aided in data analysis and manuscript revision. SA was the principal investigator who developed the concept of the study and the design, aided in data collection, and drafted and revised the manuscript. All authors contributed to the article and approved the submitted version.

## Conflict of Interest

The authors declare that the research was conducted in the absence of any commercial or financial relationships that could be construed as a potential conflict of interest.

## Publisher’s Note

All claims expressed in this article are solely those of the authors and do not necessarily represent those of their affiliated organizations, or those of the publisher, the editors and the reviewers. Any product that may be evaluated in this article, or claim that may be made by its manufacturer, is not guaranteed or endorsed by the publisher.

## Abbreviations

RT, Radiation Therapy; OARs, Organs at Risk; SBRT, Stereotactic Body Radiation Therapy; MRI, Magnetic Resonance Imaging; EM, Electromagnetic; CT, Computed Tomography; PTV, Planning Target Volume; PRV, Planning Organ at Risk Volume; BED, Biologically Effective Dose; VMAT, Volumetric Modulated Arc Therapy; FFF, Flattening Filter-Free; SD, Standard Deviation; CBCT, Cone Beam Computed Tomography; IGRT, Image Guided Radiation Therapy; CK, CyberKnife.

## References

[1] Proust-Lima C Taylor JM Sécher S Sandler H Kestin L Pickles T. Confirmation of a Low α/β Ratio for Prostate Cancer Treated by External Beam Radiation Therapy Alone Using a Post-Treatment Repeated-Measures Model for PSA Dynamics. Int J Radiat Oncol Biol Phys (2011) 79(1):195–201. doi: 10.1016/j.ijrobp.2009.10.008 20381268 PMC4122313

[2] Miralbell R Roberts SA Zubizarreta E Hendry JH. Dose-Fractionation Sensitivity of Prostate Cancer Deduced From Radiotherapy Outcomes of 5,969 Patients in Seven International Institutional Datasets: α/β = 1.4 (0.9–2.2) Gy. Int J Radiat Oncol Biol Phys (2012) 82(1):e17–24. doi: 10.1016/j.ijrobp.2010.10.075 21324610

[3] Dasu A Toma-Dasu I. Prostate α/β Revisited – an Analysis of Clinical Results From 14168 Patients. Acta Oncol (2012) 51(8):963–74. doi: 10.3109/0284186x.2012.719635 22966812

[4] Hoffman KE Voong KR Levy LB Allen PK Choi S Schlembach PJ. Randomized Trial of Hypofractionated Dose-Escalated Intensity Modulated Radiation Therapy (IMRT) Versus Conventionally Fractionated IMRT for Localized Prostate Cancer. J Clin Oncol (2018) 36(29):2943–9. doi: 10.1200/jco.2018.77.9868 PMC6804854 30106637

[5] Dearnaley D Syndikus I Mossop H Khoo V Birtle A Bloomfield D. Conventional Versus Hypofractionated High-Dose Intensity-Modulated Radiotherapy for Prostate Cancer: 5-Year Outcomes of the Randomised, Non-Inferiority, Phase 3 CHHiP Trial. Lancet Oncol (2016) 17(8):1047–60. doi: 10.1016/s1470-2045(16)30102-4 PMC4961874 27339115

[6] Catton CN Lukka H Gu CS Martin JM Supiot S Chung PWM. Randomized Trial of a Hypofractionated Radiation Regimen for the Treatment of Localized Prostate Cancer. J Clin Oncol (2017) 35(17):1884–90. doi: 10.1200/jco.2016.71.7397 28296582

[7] Lee WR Dignam JJ Amin MB Bruner DW Low D Swanson GP. Randomized Phase III Noninferiority Study Comparing Two Radiotherapy Fractionation Schedules in Patients With Low-Risk Prostate Cancer. J Clin Oncol (2016) 34(20):2325–32. doi: 10.1200/jco.2016.67.0448 PMC4981980 27044935

[8] Arcangeli S Scorsetti M Alongi F. Will SBRT Replace Conventional Radiotherapy in Patients With Low-Intermediate Risk Prostate Cancer? A Review. Crit Rev Oncol Hematol (2012) 84(1):101–8. doi: 10.1016/j.critrevonc.2011.11.009 22257653

[9] Morgan SC Hoffman K Loblaw DA Buyyounouski MK Patton C Barocas D. Hypofractionated Radiation Therapy for Localized Prostate Cancer: An ASTRO, ASCO, and AUA Evidence-Based Guideline. Pract Radiat Oncol (2018) 8(6):354–60. doi: 10.1016/j.prro.2018.08.002 30322661

[10] Kishan AU Dang A Katz AJ Mantz CA Collins SP Aghdam N. Long-Term Outcomes of Stereotactic Body Radiotherapy for Low-Risk and Intermediate-Risk Prostate Cancer. JAMA Netw Open (2019) 2(2):e188006. doi: 10.1001/jamanetworkopen.2018.8006 30735235 PMC6484596

[11] Jackson WC Silva J Hartman HE Dess RT Kishan AU Beeler WH. Stereotactic Body Radiation Therapy for Localized Prostate Cancer: A Systematic Review and Meta-Analysis of Over 6,000 Patients Treated On Prospective Studies. Int J Radiat Oncol Biol Phys (2019) 104(4):778–89. doi: 10.1016/j.ijrobp.2019.03.051 PMC6770993 30959121

[12] Widmark A Gunnlaugsson A Beckman L Thellenberg-Karlsson C Hoyer M Lagerlund M. Ultra-Hypofractionated Versus Conventionally Fractionated Radiotherapy for Prostate Cancer: 5-Year Outcomes of the HYPO-RT-PC Randomised, Non-Inferiority, Phase 3 Trial. Lancet (2019) 394(10196):385–95. doi: 10.1016/s0140-6736(19)31131-6 31227373

[13] Brand DH Tree AC Ostler P van der Voet H Loblaw A Chu W. Intensity-Modulated Fractionated Radiotherapy Versus Stereotactic Body Radiotherapy for Prostate Cancer (PACE-B): Acute Toxicity Findings From an International, Randomised, Open-Label, Phase 3, Non-Inferiority Trial. Lancet Oncol (2019) 20(11):1531–43. doi: 10.1016/s1470-2045(19)30569-8 PMC6838670 31540791

[14] Langen KM Jones DT. Organ Motion and its Management. Int J Radiat Oncol Biol Phys (2001) 50(1):265–78. doi: 10.1016/S0360-3016(01)01453-5 11316572

[15] Aubry JF Beaulieu L Girouard LM Aubin S Tremblay D Laverdière J. Measurements of Intrafraction Motion and Interfraction and Intrafraction Rotation of Prostate by Three-Dimensional Analysis of Daily Portal Imaging With Radiopaque Markers. Int J Radiat Oncol Biol Phys (2004) 60(1):30–9. doi: 10.1016/j.ijrobp.2004.02.045 15337537

[16] Kron T Thomas J Fox C Thompson A Owen R Herschtal A. Intra-Fraction Prostate Displacement in Radiotherapy Estimated From Pre- and Post-Treatment Imaging of Patients With Implanted Fiducial Markers. Radiother Oncol (2010) 95(2):191–7. doi: 10.1016/j.radonc.2010.01.010 20189667

[17] Ballhausen H Li M Hegemann NS Ganswindt U Belka C. Intra-Fraction Motion of the Prostate Is a Random Walk. Phys Med Biol (2015) 60(2):549–63. doi: 10.1088/0031-9155/60/2/549 25549204

[18] Legge K Nguyen D Ng JA Wilton L Richardson M Booth J. Real-Time Intrafraction Prostate Motion During Linac Based Stereotactic Radiotherapy With Rectal Displacement. J Appl Clin Med Phys (2017) 18(6):130–6. doi: 10.1002/acm2.12195 PMC5689913 28960696

[19] Lovelock DM Messineo AP Cox BW Kollmeier MA Zelefsky MJ. Continuous Monitoring and Intrafraction Target Position Correction During Treatment Improves Target Coverage for Patients Undergoing SBRT Prostate Therapy. Int J Radiat Oncol Biol Phys (2015) 91(3):588–94. doi: 10.1016/j.ijrobp.2014.10.049 25680601

[20] Kupelian P Willoughby T Mahadevan A Djemil T Weinstein G Jani S. Multi-Institutional Clinical Experience With the Calypso System in Localization and Continuous, Real-Time Monitoring of the Prostate Gland During External Radiotherapy. Int J Radiat Oncol Biol Phys (2007) 67(4):1088–98. doi: 10.1016/j.ijrobp.2006.10.026 17187940

[21] Zhu X Bourland JD Yuan Y Zhuang T O’Daniel J Thongphiew D. Tradeoffs of Integrating Real-Time Tracking Into IGRT for Prostate Cancer Treatment. Phys Med Biol (2009) 54(17):N393–401. doi: 10.1088/0031-9155/54/17/n03 19661570

[22] Bell LJ Eade T Kneebone A Hruby G Alfieri F Bromley R. Initial Experience With Intra-Fraction Motion Monitoring Using Calypso Guided Volumetric Modulated Arc Therapy for Definitive Prostate Cancer Treatment. J Med Radiat Sci (2017) 64(1):25–34. doi: 10.1002/jmrs.224 28263041 PMC5355366

[23] Lucchini R Panizza D Colciago RR Vernier V Daniotti MC Faccenda V. Treatment Outcome and Compliance to Dose-Intensified Linac-Based SBRT for Unfavorable Prostate Tumors Using a Novel Real-Time Organ-Motion Tracking. Radiat Oncol (2021) 16(1):180. doi: 10.1186/s13014-021-01908-0 34535168 PMC8447697

[24] Kindblom J Ekelund-Olvenmark AM Syren H Iustin R Braide K Frank-Lissbrant I. High Precision Transponder Localization Using a Novel Electromagnetic Positioning System in Patients With Localized Prostate Cancer. Radiother Oncol (2009) 90(3):307–11. doi: 10.1016/j.radonc.2008.08.018 18952311

[25] Vanhanen A Kapanen M. The Effect of Rectal Retractor on Intrafraction Motion of the Prostate Biomed. Phys Eng Express (2016) 2:35021. doi: 10.1088/2057-1976/2/3/035021

[26] Zelefsky MJ Kollmeier M Cox B Fidaleo A Sperling D Pei X. Improved Clinical Outcomes With High-Dose Image Guided Radiotherapy Compared With Non-IGRT for the Treatment of Clinically Localized Prostate Cancer. Int J Radiat Oncol Biol Phys (2012) 84:125–9. doi: 10.1016/j.ijrobp.2011.11.047 22330997

[27] de Crevoisier R Tucker SL Dong L Mohan R Cheung R Cox JD. Increased Risk of Biochemical and Local Failure in Patients With Distended Rectum on the Planning CT for Prostate Cancer Radiotherapy. Int J Radiat Oncol Biol Phys (2005) 62(4):965–73. doi: 10.1016/j.ijrobp.2004.11.032 15989996

[28] de Crevoisier R Tucker SL Dong L Mohan R Cheung R Cox JD. Daily Versus Weekly Prostate Cancer Image Guided Radiation Therapy: Phase 3 Multicenter Randomized Trial. Int J Radiat Oncol Biol Phys (2018) 102(5):1420–9. doi: 10.1016/j.ijrobp.2018.07.2006 30071296

[29] Bertholet J Knopf A Eiben B McClelland J Grimwood A Harris E. Real-Time Intrafraction Motion Monitoring in External Beam Radiotherapy. Phys Med Biol (2019) 64(15):15TR01. doi: 10.1088/1361-6560/ab2ba8 PMC7655120 31226704

[30] Gibbs IC. Frameless Image-Guided Intracranial and Extracranial Radiosurgery Using the Cyberknife Robotic System. Cancer Radiother (2006) 10(5):283–7. doi: 10.1016/j.canrad.2006.05.013 16859948

[31] Varnava M Sumida I Mizuno H Shiomi H Suzuki O Yoshioka Y. A New Plan Quality Objective Function for Determining Optimal Collimator Combinations in Prostate Cancer Treatment With Stereotactic Body Radiation Therapy Using CyberKnife. PloS One (2018) 13(11):e0208086. doi: 10.1371/journal.pone.0208086 30481228 PMC6258559

[32] Masi L Zani M Doro R Calusi S Di Cataldo V Bonucci I. L. CyberKnife MLC-Based Treatment Planning for Abdominal and Pelvic SBRT: Analysis of Multiple Dosimetric Parameters, Overall Scoring Index and Clinical Scoring. Phys Med (2018) 56:25–33. doi: 10.1016/j.ejmp.2018.11.012 30527086

[33] Giżyńska MK Rossi L den Toom W Milder MTW de Vries KC Nuyttens J. Largely Reduced OAR Doses, and Planning and Delivery Times for Challenging Robotic SBRT Cases, Obtained With a Novel Optimizer. J Appl Clin Med Phys (2021) 22(3):35–47. doi: 10.1002/acm2.13172f PMC7984474 33475227

[34] Shelton J Rossi PJ Chen H Liu Y Master VA Jani AB. Observations on Prostate Intrafraction Motion and the Effect of Reduced Treatment Time Using Volumetric Modulated Arc Therapy. Pract Radiat Oncol (2011) 1(4):243–50. doi: 10.1016/j.prro.2011.02.008 24674002

[35] Li JS Lin MH Buyyounouski MK Horwitz EM Ma CM. Reduction of Prostate Intrafractional Motion From Shortening the Treatment Time. Phys Med Biol (2013) 58(14):4921–32. doi: 10.1088/0031-9155/58/14/4921 PMC3940444 23798642

[36] Mah D Freedman G Milestone B Hanlon A Palacio E Richardson T. Measurement of Intrafractional Prostate Motion Using Magnetic Resonance Imaging. Int J Radiat Oncol Biol Phys (2002) 54(2):568–75. doi: 10.1016/s0360-3016(02)03008-0 12243837

[37] Quon H Loblaw DA Cheung PC Holden L Tang C Pang G. Intra-Fraction Motion During Extreme Hypofractionated Radiotherapy of the Prostate Using Pre- and Post-Treatment Imaging. Clin Oncol (R Coll Radiol) (2012) 24(9):640–5. doi: 10.1016/j.clon.2011.12.001 22226683

[38] Koike Y Sumida I Mizuno H Shiomi H Kurosu K Ota S. Dosimetric Impact of Intra-Fraction Prostate Motion Under a Tumour-Tracking System in Hypofractionated Robotic Radiosurgery. PloS One (2018) 13(4):e0195296. doi: 10.1371/journal.pone.0195296 29621319 PMC5886484

[39] Gorovets D Burleson S Jacobs L Ravindranath B Tierney K Kollmeier M. Prostate SBRT With Intrafraction Motion Management Using a Novel Linear Accelerator-Based MV-kV Imaging Method. Pract Radiat Oncol (2020) 10(5):e388–96. doi: 10.1016/j.prro.2020.04.013 32454176

[40] Levin-Epstein R Qiao-Guan G Juarez JE Shen Z Steinberg ML Ruan D. Clinical Assessment of Prostate Displacement and Planning Target Volume Margins for Stereotactic Body Radiotherapy of Prostate Cancer. Front Oncol (2020) 10:539. doi: 10.3389/fonc.2020.00539 32373529 PMC7177009

[41] Wolf J Nicholls J Hunter P Nguyen DT Keall P Martin J. Dosimetric Impact of Intrafraction Rotations in Stereotactic Prostate Radiotherapy: A Subset Analysis of the TROG 15.01 SPARK Trial. Radiother Oncol (2019) 136:143–7. doi: 10.1016/j.radonc.2019.04.013 31015116

[42] Zhang P Hunt M Happersett L Yang J Zelefsky M Mageras G. Robust Plan Optimization for Electromagnetic Transponder Guided Hypo-Fractionated Prostate Treatment Using Volumetric Modulated Arc Therapy. Phys Med Biol (2013) 58(21):7803–13. doi: 10.1088/0031-9155/58/21/7803 24145674

[43] Braide K Lindencrona U Welinder K Götstedt J Ståhl I Pettersson N. Clinical Feasibility and Positional Stability of an Implanted Wired Transmitter in a Novel Electromagnetic Positioning System for Prostate Cancer Radiotherapy. Radiother Oncol (2018) 128(2):336–42. doi: 10.1016/j.radonc.2018.05.031 29921461

[44] Vanhanen A Syrén H Kapanen M. Localization Accuracy of Two Electromagnetic Tracking Systems in Prostate Cancer Radiotherapy: A Comparison With Fiducial Marker Based Kilovoltage Imaging. Phys Med (2018) 56:10–8. doi: 10.1016/j.ejmp.2018.11.007 30527084

